# Functional Impact of Missense Variants in BRCA1 Predicted by Supervised Learning

**DOI:** 10.1371/journal.pcbi.0030026

**Published:** 2007-02-16

**Authors:** Rachel Karchin, Alvaro N. A Monteiro, Sean V Tavtigian, Marcelo A Carvalho, Andrej Sali

**Affiliations:** 1 Department of Biomedical Engineering, Johns Hopkins University, Baltimore, Maryland, United States of America; 2 Institute of Computational Medicine, Johns Hopkins University, Baltimore, Maryland, United States of America; 3 Risk Assessment, Detection, and Intervention Program, H. Lee Moffitt Cancer Center and Research Institute, Tampa, Florida, United States of America; 4 International Agency for Research on Cancer, Lyon, France; 5 Department of Pharmaceutical Chemistry, University of California San Francisco, San Francisco, California, United States of America; 6 California Institute for Quantitative Biomedical Research, University of California San Francisco, San Francisco, California, United States of America; Lilly Systems Biology, Singapore

## Abstract

Many individuals tested for inherited cancer susceptibility at the *BRCA1* gene locus are discovered to have variants of unknown clinical significance (UCVs). Most UCVs cause a single amino acid residue (missense) change in the BRCA1 protein. They can be biochemically assayed, but such evaluations are time-consuming and labor-intensive. Computational methods that classify and suggest explanations for UCV impact on protein function can complement functional tests. Here we describe a supervised learning approach to classification of *BRCA1* UCVs. Using a novel combination of 16 predictive features, the algorithms were applied to retrospectively classify the impact of 36 BRCA1 C-terminal *(BRCT)* domain UCVs biochemically assayed to measure transactivation function and to blindly classify 54 documented UCVs. Majority vote of three supervised learning algorithms is in agreement with the assay for more than 94% of the UCVs. Two UCVs found deleterious by both the assay and the classifiers reveal a previously uncharacterized putative binding site. Clinicians may soon be able to use computational classifiers such as those described here to better inform patients. These classifiers can be adapted to other cancer susceptibility genes and systematically applied to prioritize the growing number of potential causative loci and variants found by large-scale disease association studies.

## Author Summary

A significant number of breast and ovarian cancers are due to inherited mutations in the BRCA1 and BRCA2 genes. Many women who receive genetic testing for these mutations are found to have variants of the genes that result in changed amino acids in the BRCA1 or BRCA2 proteins. The effect of these variants on cancer risk is not well-understood, posing a problem for patients and their health providers. We describe computational biology methods that predict and analyze the impact of 36 BRCA1 variants on protein function. The predictions are validated by biochemical assays of BRCA1 in yeast and mammalian cell cultures. The speed and accuracy of the computational methods is well-suited to rapid evaluation of large numbers of variants in genes that predispose to inherited diseases.

## Introduction

The *BRCA1* gene encodes a large multifunction protein involved in cell-cycle and centrosome control, transcriptional regulation, and in the DNA damage response [1–3]. Inherited mutations in this gene have been associated with an increased lifetime risk of breast and ovarian cancer (6–8 times that of the general population) [4]. There are several thousand known deleterious *BRCA1* mutations that result in frameshifts and/or premature stop codons, producing a truncated protein product [5]. In contrast, the functional impact of most missense variants that result in a single amino acid residue change in BRCA1 protein is not known. The Breast Cancer Information Core database (http://research.nhgri.nih.gov/bic/), a central repository of *BRCA1* and *BRCA2* mutations identified in genetic tests, currently contains 487 unique missense *BRCA1* variants (April 2006), of which only 17 have sufficient genetic/epidemiological evidence to be classified as deleterious (Clinically Important) and 33 as neutral or of little clinical importance (Not Clinically Important). As genetic testing for inherited disease predispositions becomes more commonplace, predicting the clinical significance of missense variants and other UCVs will be increasingly important for risk assessment.

Because most UCVs in *BRCA1* and *BRCA2* occur at very low population frequencies (<0.0001) [6], direct epidemiological measures, such as familial cosegregation with disease, are often not sufficiently powerful to identify the variants associated with cancer predisposition. A promising approach is to supplement epidemiological and clinical analysis of UCVs with indirect approaches such as biochemical studies of protein function and bioinformatics analysis [6–8]. In the future, physicians and genetic counselors may be able to rely on all these sources of information about UCVs when counseling their patients.

Previous bioinformatics analysis of *BRCA1* UCVs has depended primarily on measures of evolutionary conservation in multiple sequence alignments of human *BRCA1* and related proteins from other organisms [9–11]. Two groups have attempted to include information about BRCA1 protein structure. Williams et al. predicted the impact of 25 missense variants in BRCA1′s C-terminal BRCT domains by considering both conservation and location of variant amino acid residues in an X-ray crystal structure [12]. Variants were predicted deleterious if their properties were similar to properties of biochemically characterized deleterious variants in Escherichia coli Lac Repressor and bacteriophage T4 lysozyme. Mirkovic et al. developed a set of hierarchical rules (Rule-based decision tree) based on the conservation, variant structural location, and amino acid residue physiochemical properties of 30 deleterious and seven neutral biochemically characterized BRCA1 missense variants [7].

We have developed a novel combination of 16 predictive features that describe conservation, impact of mutation on protein structure, and amino acid residue properties, and used them as input to computational supervised learning algorithms. These algorithms are trained to learn a generic classification of amino acid residue substitutions and positional contexts. The training set is composed of 618 missense variants in the transcription factor TP53 biochemically characterized as functional or nonfunctional in a transactivation assay [13]. TP53 is a tumor suppressor gene that is inactivated in the majority of human cancers.

Our validation set is composed of 36 missense variants in BRCA1′s BRCT domains that were biochemically characterized with a transactivation assay [14]. These 36 variants were selected because they occur in individuals from families with breast or ovarian cancer in which no other deleterious mutation in *BRCA1* or *BRCA2* was found and were functionally tested under the same protocols and conditions, yielding standardized measurements of each variant's transactivation activity with respect to wild-type. We use the validation set to assess the supervised learners and compare them with algorithms based on evolutionarily allowed amino acid residues or empirically derived rules. The algorithms with greatest correlation between assay and computational predictions are the supervised learners Naïve Bayes [15], Support Vector Machine [16], and Random Forest [17].

Given a protein X-ray crystal structure, the supervised learning approach can quickly and accurately predict the outcome of our BRCA1 transactivation assay with greater than 94% accuracy on tested missense variants in the BRCT domains. We have applied the best performing supervised learners to blind prediction of the functional impact of 54 UCVs found in BIC and occurring in the BRCA1 BRCT domains. For each of these UCVs, we produce a consensus prediction and, where possible, a molecular explanation for the impact of the variant.

Next, we describe the protocol used to train and validate the supervised learning algorithms, the selection of 16 features used to represent each missense variant to the algorithms, implementation details of each algorithm, and performance assessment criteria (Methods). We then show how a combination of sequence- and structure-based features in a supervised learning setting obviates some of the problems with evolutionary analysis and empirically derived rules, providing specific examples of the strengths and weaknesses of each approach (Results, Discussion). We show that two of the variants found to be deleterious by both the assay and the classifiers may be at a previously uncharacterized protein binding site and that electrostatic changes at the site may weaken the interactions of BRCA1 and protein partners that are important for its functions (Discussion). Finally, we discuss the generalizability of our methods to other cancer susceptibility genes and to large-scale disease association studies (Discussion).

## Methods

### Training Set

We trained four supervised learning algorithms to discriminate between a set of 398 deleterious/nonfunctional and 220 neutral/functional TP53 missense variants, biochemically characterized in a transactivation assay [13]. The variants were downloaded from the IARC TP53 website (http://www-p53.iarc.fr). We only used variants capable or incapable of activating transcription for all eight of the TP53 promoters tested in the transactivation assay and located in the core DNA binding domain of TP53.

### Validation Set

The 36 BRCA1 BRCT missense variants described in our companion paper [14] were used as an independent validation set for the supervised learners. These variants were also classified by sequence-analysis methods based on evolutionarily allowed amino acid residues: Align Grantham Variation Grantham Deviation (Align-GVGD) [18], Sorting Intolerant from Tolerant (SIFT) [19], Ancestral Sequence [9,11], and empirically derived rules encoded in a decision tree (Rule-based decision tree) [7]. Each method was evaluated by its agreement with the BRCA1 transactivation assay on the validation set, according to accuracy (fraction of all variants correctly classified), sensitivity or true positive rate (fraction of all nonfunctional variants correctly classified), specificity or true negative rate (fraction of all functional variants correctly classified), Matthews correlation coefficient [20], and coverage (fraction of variants for which a prediction was made) (Table 1). Matthews correlation coefficient is defined as

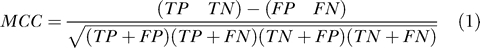
and ranges from *−1.0* (worst) to *1.0* (best). A coefficient of *0* is equivalent to a random prediction, and less than *0* indicates a worse than random prediction. *TP* is the number of correctly classified nonfunctional variants, *TN* the number of correctly classified functional variants, *FP* the number of incorrectly classified nonfunctional variants, and *FN* the number of incorrectly classified functional variants.

**Table 1:**
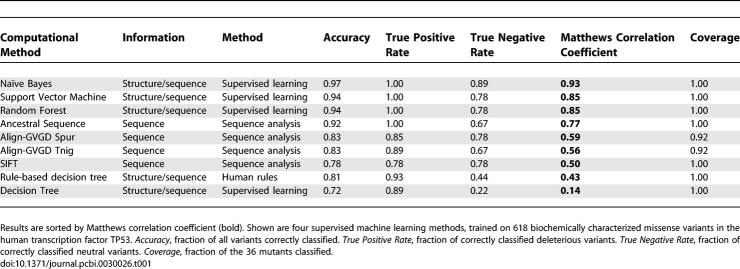
Classification Performance of Nine Computational Methods for UCV Classification, According to Agreement with the BRCA1 Transactivation Assay of 36 Missense Variants

For the Naïve Bayes, Support Vector Machine, Random Forest, Decision Tree, Align-GVGD, and SIFT classifiers, we computed a receiver operating characteristic (ROC) curve that quantifies the tradeoff between coverage of detected nonfunctional variants *(true positive rate)* and misclassified functional variants *(false positive rate = 1* − *specificity)*. ROC analysis was not possible for the Rule-based decision tree and Ancestral Sequence algorithms, which predict the class of a missense variant but do not provide an associated score.

### Feature Selection

The supervised learning algorithms (Naïve Bayes, Support Vector Machine, Random Forest, Decision Tree) were trained by associating each amino acid residue substitution in the TP53 training set with 16 carefully selected predictive features (Table 2). A vector of features for a single substitution is denoted as 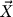 . The features describe properties of variant and wild-type residues: local structural environment; physiochemical attributes; and evolutionary conservation. To compute the features, we used DSSP (a program that calculates a variety of geometrical properties for each amino acid residue in a protein structure) [21], MODELLER for comparative protein structure modeling [22], SAM-T2K for protein sequence alignments and hidden Markov models [23], and in-house PERL code.

**Table 2:**
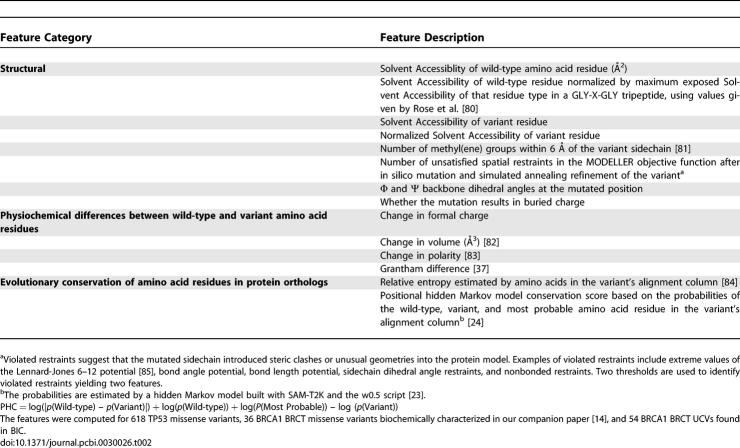
Predictive Features Describing Evolutionary Conservation, Impact of Mutation on Protein Structure, and Amino Acid Residue Properties Used as Input to the Computational Supervised Learning Algorithms

We began with a core set of 13 features selected by a correlation analysis between features and classes (functional or nonfunctional) of the TP53 variants, as described previously [24] (Table S3). An additional 18 candidate features were evaluated by adding them to the core set and doing 10-fold cross-validation tests of Support Vector Machine performance. Three features were found to improve performance and were added to the optimal feature set; the others were rejected (Tables S4 and S5). Next we evaluated Support Vector Machine performance with each of the best 16 features held out. In each case, the 10-fold cross-validation test yielded decreased performance.

### Protein Modeling

We used X-ray crystal structures from the Protein Data Bank [25] for the BRCA1 BRCT domains (1t29 chain A in complex with BACH1 peptide) [26] and the DNA binding domain of TP53 (1kzy) [27]. We performed in silico mutations on the structures with the MUTATE_MODEL routine of MODELLER (available as a Python script at http://salilab.org/modeller/wiki/Mutate_model). MUTATE_MODEL substitutes the wild-type amino acid residue at a position of interest with a variant amino acid residue, and optimizes the coordinates of the variant's backbone and sidechain atoms with an initial conjugate gradient minimization, molecular dynamics optimization with simulated annealing, and a final conjugate gradient minimization (E. Feyfant, 2004, private communication).

### Protein Sequence Alignments

The amino acid residue sequences of human TP53 (P04637) and BRCA1 (P38398) were downloaded from UNIPROT [28], and each was used as a seed sequence for the SAM-T2K iterative alignment-building algorithm [23]. For BRCA1, only amino acid residues in the BRCT domains (1649–1859) were aligned. We used the SAM w0.5 program to apply sequence weighting and regularization with Dirichlet mixtures [29] to each resulting alignment and to produce a profile hidden Markov model [30]. The TP53 and BRCA1 alignments and hidden Markov models are available upon request.

### Support Vector Machine

We trained a soft margin Support Vector Machine classifier with a radial basis kernel using the e1071 package in R [31]. The Support Vector Machine algorithm optimizes a vector of weights 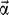 (one weight for each training example) and a bias parameter *b*. The parameters *g* (radial basis kernel width) and *C* (penalty for violating the soft margin) were optimized on the training set with grid search using default parameters. Each of the 28 missense variants 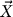 was then scored with the discriminant function

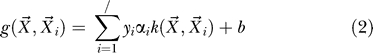
where *l* is the number of examples in the training set, *y_i_* is the class label of each example in the training set (for deleterious/nonfunctional variants *y_i_ =* −*1* and for neutral/functional variants *y_i_ = 1*), and 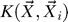 is the value of the radial basis kernel function given 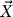 and training example 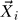 . Variants are classified as deleterious/nonfunctional if 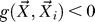 and neutral/functional if 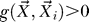 .

### Naïve Bayes

The Naïve Bayes algorithm estimates the probability that each variant belongs to deleterious or neutral classes 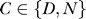 by applying the Bayes rule:

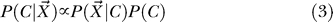
where the prior class probability *P*(*C*) is the fraction of deleterious (or neutral) missense variants in the training set and each feature *X_i_* is assumed to be conditionally independent of the *k − 1* other features, given its class membership, so that

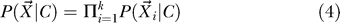
where *P*(*X_i_* | *C*) is estimated from the training set. We used the Naïve Bayes method in R's e1071 package. Each feature was approximated to be normally distributed and no smoothing was applied to the feature distributions.

### Decision Tree

We used the rpart package in R [32] to train a Decision Tree with the following parameters: *minsplit = 20* (minimum number of observations required at a tree node before a split is attempted) and *cp = 0* (no pruning of tree regardless of whether a split will improve model fit). To reduce overfitting, we pruned the resulting tree using the standard heuristic “1 Standard Error rule” [33] and 10-fold cross-validation. According to the 1 Standard Error rule, the pruned tree with best generalization properties has a cross-validation error on the training set 1 Standard Error worse than the tree with the lowest cross-validation error. The pruning process yielded a reduced set of features: Φ and Ψ mainchain dihedral angles, normalized solvent accessibility of wild-type, Grantham difference, volume change, relative entropy, and positional hidden Markov model conservation score.

### Random Forest

We used the randomForest package in R [34] to train a Random Forest, an algorithm based on a majority vote of a large number of decision trees, in which the candidate features at each tree node are randomly sampled [17]. The user-defined input parameters to randomForest are total number of trees in the forest and *mtry* (number of randomly sampled features considered as candidates for a split at each tree node). Both were selected with grid-search optimization as described for the Support Vector Machine [31].

### Log Likelihood Ratios

Predictions of Naïve Bayes, Decision Tree, and Random Forest are in the form of class conditional probabilities, where the two classes are *D* (deleterious/nonfunctional) and *N* (neutral/functional). For each example, the classifiers report *P*(*D |* 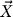 ) (probability that the variant is deleterious, given feature vector 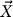 ) and *P*(*N |* 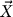 ) (probability that the variant is neutral, given feature vector 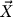 ). To evaluate accuracy, true positive rate, true negative rate, and Matthews correlation coefficient, we classified variants as deleterious if *P*(*D |* 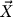 ) *> 0.5* and neutral otherwise. To compute ROC curves, we used the log likelihood ratio

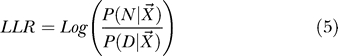
as the output score of Naïve Bayes, Decision Tree, and Random Forest.

### SIFT

The SIFT algorithm [19] predicts the probability that a missense mutation occurs at a given alignment position. Variants that occur at conserved alignment positions are expected to be tolerated less than those that occur at diverse positions. The algorithm uses a modified version of PSI-BLAST [35] and Dirichlet mixture regularization [29] to construct a multiple sequence alignment of proteins that can be globally aligned to the query sequence and belong to the same clade. We used the SIFT server (http://blocks.fhcrc.org/sift/SIFT.html), with PSI-BLAST search set to the Swissprot-TrEMBL protein sequence database [36]. Both the full-length human BRCA1 sequence (amino acid residues 1–1863) and the BRCT C-terminal domain sequence only (amino acid residues 1649–1859) were submitted to the server. For the full-length sequence, SIFT reported low confidence predictions for 34 out of 36 missense variants. Consequently, we based our SIFT predictions on the C-terminal domain sequence. To compute accuracy, true positive rate, true negative rate, and Matthews correlation coefficient, we used the binary class predictions of the SIFT server (deleterious or neutral), based on the default SIFT threshold (*tolerated mutation probability > 0.05*). For ROC analysis, we used the raw SIFT probabilities, which range from 0 to 1.

### Ancestral Sequence

The Ancestral Sequence classifications were computed as described [9,11]. Each position in an alignment of eight mammalian *BRCA1* orthologs identified as giving best results by Pavlicek et al. was categorized as fixed (completely conserved), conserved (substitution of similar amino acid residues), or nonconserved (dissimilar amino acid residues or gaps). Any substitution at a fixed position and any nonconservative substitution at a conserved position is classified as deleterious. Amino acid residue similarity is based on the Gonnet PAM250 score (i.e.*,* the likelihood that amino acid residue A has mutated into amino acid residue B in a pair of sequences that have diverged by 250 mutations per 100 amino acid residues of sequence) [37].

### Align-GVGD

The Align-GVGD method calculates two scores for each amino acid residue substitution, Grantham Deviation (GD) and Grantham Variation (GV), based on a modified Grantham distance measure [18,38]. The scores define four categories of missense variants: “Enriched deleterious 1” variants occur at invariant alignment positions for which the substitution is outside the range of variation observed at the position (GV = 0, GD > 0); “Enriched deleterious 2” occur at variable alignment positions containing physiochemically similar amino acid residues where the substitution is outside the range of observed variation (0 < GV < 61.3, GD = 0); “Enriched neutral 1” occur at variable positions containing physiochemically similar amino acid residues where the substitution is inside the range of variation (GV > 0, GD = 0); and “Enriched neutral 2” occur at variable positions containing dissimilar amino acid residues where the substitution is slightly outside the range of variation (GV > 61.3, 0 < GD < 61.3). We classified variants using first an alignment of placental mammals, a marsupial (gray short-tailed opposum), chicken, frog, and the pufferfish *Tetraodon* (“Align-GVGD Tnig”), and second an alignment that also includes the sea urchin Strongylocentrotus purpuratus (“Align-GVGD Spur”). Accuracy, true positive rate, true negative rate, and Matthews correlation coefficient were evaluated by reducing the four categories to deleterious/nonfunctional or neutral/functional. Variants may have GV and GD values that do not match any of the four categories (e.g., a variant with GV = 80 and GD = 80), which lowers coverage, Matthews correlation coefficient, true positive, and false positive rates. For ROC analysis, rather than fixing thresholds on GV and GD at 61.3 for each substitution, we considered the number of true positives and false positives over a range of thresholds, from the smallest to largest values of GV and GD in our dataset (0 to 215).

### Rule-Based Decision Tree

A Rule-based decision tree is a classification tree with human-designed rules that uses both structure- and sequence-based information, implemented in PERL [7]. Rule-based Decision Tree classifies a missense variant as either deleterious/nonfunctional or neutral/functional, but does not compute numerical scores.

### Structural Variant Analysis

The structural models of all BRCA1 BRCT missense variants were visually compared with the wild-type structure (1t29) using the molecular graphics program Chimera [39]. We explored changes in hydrogen bonding patterns and geometric properties of the molecular surface with Chimera's FindHBond and MSMS routines. To visualize the distribution of amino acid residue conservation on the protein surface, the RenderByAttribute routine was used, with coloration defined by percent conserved in a hand-edited SAM-T2K alignment of BRCA1 orthologs. Species used in this alignment were Homo sapiens (AAA 73985), Pan troglodytes (AAG43492), Gorilla gorilla (AAT44835), Pongo pygmaeus (AAT44834), Macaca mullata (AAT44833), Canis familiaris (AAC48663), Bos taurus (AAL76094), Monodelphis domestica (AAX92675), Mus musculus (AAD00168), Rattus norvegicus (AAC36493), Gallus gallus (AAK83825), Xenopus laevis (AAL13037), and Tetraodon nigroviridis (AAR89523).

A highly conserved surface patch was identified as a possible binding site and subjected to further analysis. We used DELPHI [40] to compute the electrostatic surface potential at the putative binding site for the wild-type structure and for models of two solvent-exposed variants characterized as deleterious in our functional assays (T1685A and R1753T) [14]. The solvent relative dielectric constant was set to 4.0, the protein relative dielectric constant to 20.0, and ionic strength to the physiological value of 0.2 mM. Charges were estimated with the united atom AMBER model [41]. The proteins were prepared for DELPHI by adding heavy atoms missing from the 1t29 crystal structure with MODELLER's COMPLETE_PDB routine and adding hydrogens with REDUCE [42], then visualized in Chimera with a GRASP surface representation [43].

## Results

**Figure 1:**
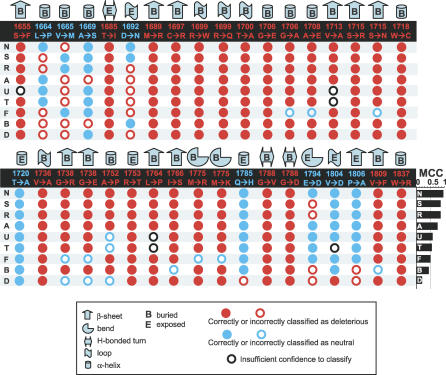
Computational Classifications of 36 BRCA1 BRCT Variants Functionally Characterized by the Transactivation Assay For each variant, the local protein structure environment is represented by secondary structure type and whether the amino acid residue is buried (*normalized solvent accessibility < 0.2*) or exposed (*normalized solvent accessibility ≥ 0.2*). Labels (“1655 S->F”) are colored according to whether the variant was functional in the assays (blue) or nonfunctional (red). Computational classifications in agreement with the assay are indicated by filled circles. Computational classifications not in agreement with the assay are indicated by outlined circles. Computational classifications yielding “unclassified” are indicated by an outlined black circle. The variant D1692N is fully functional as a transcriptional activator but results in incorrect splicing in vivo. Results from variant M1775K are unpublished (Foulkes et al.). A, Ancestral Sequence; B, Rule-based decision tree; D, Decision Tree; F, SIFT; MCC, Matthews correlation coefficient; N, Naïve Bayes; R, Random Forest; S, Support Vector Machine; T, Align-GVGD Tnig; U, Align-GVGD Spur.

We compared the transactivation activity of the wild-type LexA DBD-BRCA1 or GAL4DBD-BRCA1 fusion construct in both yeast and mammalian cells with the activity of constructs containing 36 single missense variants in the BRCT domains [14]. Variant constructs presenting 50% or more of wild-type activity are characterized as neutral and those with 45% or less are characterized as deleterious, thresholds that are in agreement with available genetic evidence. These functional characterizations were used as a standard to evaluate the reliability of nine computational classifiers (Figure 1). We also provide a post-prediction analysis of these variants (Table S1). Three classifiers with the highest correlation to the functional assay were applied to predict the impact of 54 UCVs in the BRCT domains currently listed in the Breast Information Core database.

### Algorithm Evaluation

**Figure 2:**
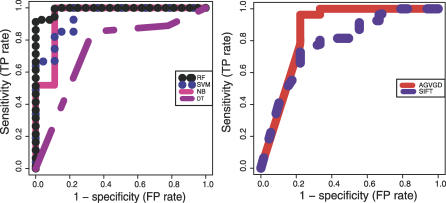
Sensitivity versus 1-Specificity of Classifiers That Use a Numerical Score to Predict the Functional Impact of 34 BRCA1 BRCT UCVs Comparison of four supervised machine learning methods, trained on 618 biochemically characterized missense variants in the human transcription factor TP53 and two sequence analysis methods that consider evolutionary conservation and physiochemical properties of amino acids (SIFT and Align-GVGD Tnig based on alignment of eight placental mammals, marsupial, chicken, frog, and pufferfish). Align-GVGD Spur, using an alignment that includes these species plus sea urchin, performs slightly worse than Align-GVGD Tnig in terms of ROC analysis and is not shown. Plot created with ROCR [86]. DT, decision tree; NB, Naïve Bayes; RF, random forest; SVM, support vector machine.

Based on ROC analysis, the supervised learners Random Forest, Support Vector Machine, and Naïve Bayes yield the most reliable computational classifications of the 36 variants (Figure 2). The area under the ROC curve (AUC) quantifies the probability that a classifier will give a randomly drawn deleterious example a lower score than a randomly drawn neutral example. AUC is 0.992 for Random Forest, 0.947 for Support Vector Machine and Naïve Bayes, 0.86 for Align-GVGD Tnig, 0.852 for Align-GVGD Spur, 0.783 for SIFT, and 0.738 for Decision Tree (Figure 2). The Decision Tree algorithm appears to overfit the training set and generalizes less well than the other supervised learners.

Three of the supervised learning algorithms (Naïve Bayes, Support Vector Machine, and Random Forest) produce the best classifications of the 36 variants, as measured by accuracy, true positive rate, true negative rate, Matthews correlation coefficient, and coverage, using default thresholds (Table 1). According to these statistical measures, the best sequence analysis methods are Ancestral Sequence and Align-GVGD. Random Forest, Naïve Bayes, and Support Vector Machine are the most accurate scoring predictors, according to the AUC. The methods rankings are slightly different when evaluated by threshold-dependent statistics that reduce predictive scores to deleterious/neutral classes or by the score-based and threshold-independent ROC statistic of AUC.

### BIC BRCA1 UCVs in BRCT Domains

**Figure 3:**
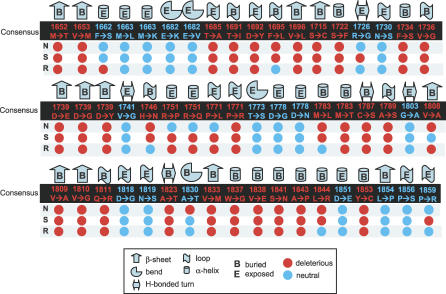
Computational Classifications of 54 Uncharacterized Variants Found in BIC For each variant, the local protein structure environment is represented by secondary structure type and whether the amino acid residue is buried (*normalized solvent accessibility < 0.2*) or exposed (*normalized solvent accessibility ≥ 0.2*). For the 54 uncharacterized variants, labels (“1652 M->T”) are colored according to consensus prediction from Naïve Bayes, Support Vector Machine, and Random Forest. Predictions of each method are indicated by filled circles (blue, neutral; red, deleterious). N, Naïve Bayes. R, Random Forest; S, Support Vector Machine.

We applied the top performing algorithms (Naïve Bayes, Support Vector Machine, and Random Forest) to predict the impact of 54 BRCA1 UCVs listed in BIC that (a) are located in the BRCT domains, and (b) have not been functionally characterized by our transactivation assays. Based on a majority vote of the computational predictors, we computed a “consensus prediction” for each UCV (Figure 3). We provide structural explanations for the impact of as many variants as possible, and indicate where the predictions are supported by biochemical experiments found in the literature (Table S2).

**Figure 4:**
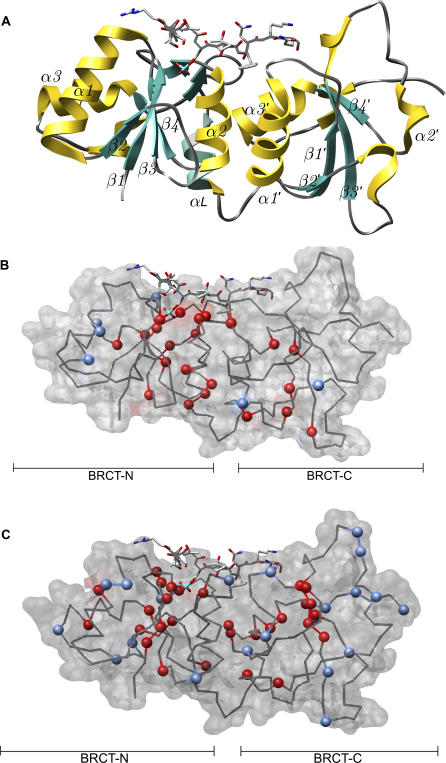
Spatial Distribution of Predicted Deleterious and Neutral Missense Variants in the BRCA1 BRCT Domains (A) Ribbon representation of the two domains with labeled helices (α1, α2, etc.) and strands (β1, β2, etc.). Recreation of Figure 1A [64]. (B) BRCA1 BRCT missense variants reported as neutral (blue) and deleterious (red) in the mammalian transactivation assay shown mapped onto the BRCA1 BRCT X-ray crystal structure (1t29). (C) Consensus predictions of Random Forest, Naïve Bayes, and Support Vector Machine for 54 BRCA1 BRCT VUS in the Breast Information Core database (http://research.nhgri.nih.gov/bic/BIC/) mapped onto the same structure, with predicted neutral shown in blue and predicted deleterious in red.

The predicted deleterious UCVs are predominantly in the core secondary structure elements of the BRCT domains, rather than in loops, particularly in the β sheet of BRCT-N (β1, β3, and β4), and in helix α′3 and the turn connecting helix α′1 and strand β′2 in BRCT-C (Figure 4).

### Binding-Site Prediction

**Figure 5:**
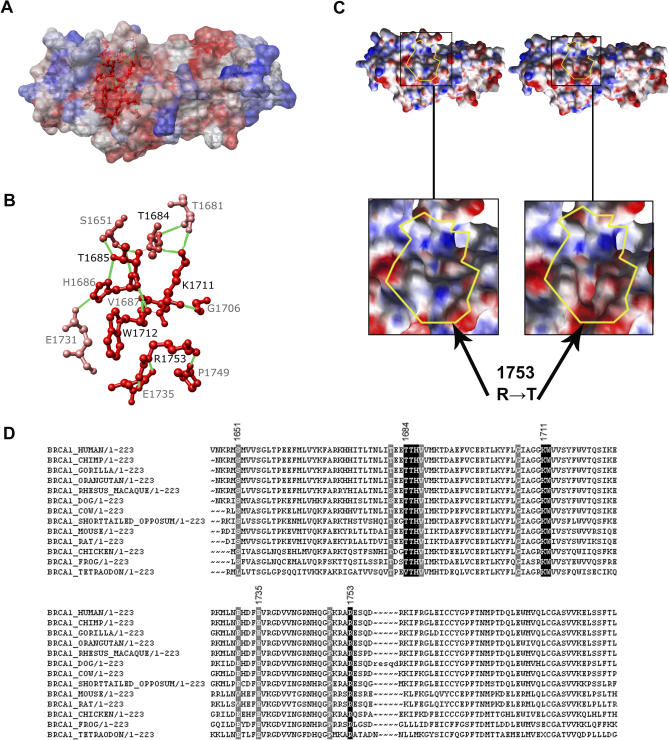
Identification of a Putative Novel Binding Site in BRCA1 BRCT Domains Two surface variants found to be deleterious to BRCA1 activity in our companion paper (R1753T and T1685I) [14] lie at a highly conserved patch of amino acid residues, forming a groove on the protein surface, possibly a heretofore uncharacterized binding site of BRCA1 with a protein partner or nucleotide ligand. (A) Surface representation of BRCA1 BRCT domains colored by conservation in our multiple sequence alignment of orthologs. Red, 100% conserved; white, 39% conserved; blue, 0% conserved. (B) Two hydrogen-bonding networks are shown in ball-and-stick format. (C) Changes in the electrostatic surface potential of the putative binding site upon mutation of R1753 to T1753. The electrostatic surface potential of the groove changes from primarily positive (greater than 10 *k*T) and neutral (0 *k*T), depicted as blue and white, to negative (less than −10 *k*T), depicted as red. This change may weaken the binding of protein partner(s) or nucleic acid ligand(s) necessary for BRCA1′s transactivation activity. Electrostatic surface potential calculated by DELPHI, visualized by CHIMERA in GRASP format [39,40,43]. (D) Multiple sequence alignment of BRCT domains in BRCA1 orthologs. Primary groove residues are shaded in black, and their hydrogen-bonding partners are shaded in gray.

We observed a patch of highly conserved amino acid residues that form a groove on the BRCA1 surface, on the opposite face from the known phosphopeptide binding cleft (Figure 5A–5C). These residues are T1684, T1685, H1686, K1711, W1712, and R1753. Both T1685 and H1686 have been shown to be highly sensitive to mutation, and our companion paper [14] contains new experimental evidence that R1753T has markedly reduced transactivation activity in both yeast and mammalian cells. The groove residues form hydrogen bonds with each other and several other conserved residues, including S1651, V1687, T1681, G1706, E1731, E1735, and P1749, producing two hydrogen bonding networks. The first network is found in BRCT-N (S1651, T1684, T1685, H1686, V1687, G1706, K1711, W1712, E1731) and the second network connects BRCT-N residues with the linker region that connects BRCT-N and BRCT-C (E1735, P1749, R1753) (Figure 5A–5C). All the residues lining this groove are completely conserved in our alignment of *BRCA1* orthologs, except for T1684, which is conserved in all orthologs except for *Tetraodon* (pufferfish), the organism most distant from human in our alignment (Figure 5A). Previous studies have shown that G1706 and P1749 are also sensitive to mutation [44,45] (S. Marsillac, 2006, private communication). The proposed binding site would be specific to BRCA1, as most of these positions (except for H1686) are not highly conserved across tandem BRCT repeats in MDC1, PTIP, BARD1, and 53BP1.

The solvent-exposed missense variant R1753T found at the proposed binding site has <20% of the wild-type transactivation activity in yeast and <5% in mammalian cells [14], suggesting that the wild-type arginine amino acid residue might be important for binding of BRCA1 to a protein partner (or nucleic acid ligand). Although the mechanism of the BRCA1 BRCT domains in transactivation is not known, it is believed to depend on interactions with a variety of partners [46,47]. The mutation of R1753 to a threonine changes the local electrostatic surface potential from primarily positive and neutral (depicted as blue and white) to negative (red) (Figure 5D). This change may weaken the binding of protein partner(s) or nucleic acid ligand(s) necessary for transactivation.

## Discussion

We have developed an approach to rapid characterization of inherited missense variants in the BRCT domains of *BRCA1* that is able to retrospectively predict the outcome of a functional transactivation assay with greater than 94% accuracy. Our method makes no a priori assumptions about which predictive features might best inform a classification algorithm. Rather, we hypothesize that given a large sample of deleterious and neutral variants, we can quantitatively measure the most informative predictors and learn to distinguish between these two functional classes with a supervised learning algorithm. We discuss (a) how our approach compares with similar work [7,12,18,19,48–50], (b) how prediction of deleterious variants can identify putative binding sites, (c) how computational classifiers can save time and money required for biochemical assays of many candidate variants, and (d) the possibilities of generalizing the methods to large numbers of disease-associated genes.

Supervised learning algorithms have previously been applied to predicting the functional impact of missense variants in TP53 with a four-body “potential” based on Delauney tessellation [51], to engineered variants in *E. Coli* Lac Repressor, HIV protease, and T4 bacteriophage lysozyme [24,52,53], and to large sets of single nucleotide polymorphisms [54–56]. Much of this work has been limited by overfitting problems. Benchmarking of Support Vector Machines and Decision Trees in several studies has shown that high numbers of false positive and false negative classification errors (~0.30) are generated when the learners are applied to proteins other than those in their training sets [24,52]. Supervised learning using four-body potentials is further limited in application, because each missense variant is represented by a profile of *n* features (amino-acid residue potential scores), where *n* is the number of amino acid residues in the protein. Supervised learning algorithms require fixed-length feature vectors; thus, an algorithm trained on missense variants represented by *n* features can only classify missense variants that are represented by *n* features. For example, if the training set is composed of missense variants in Lac Repressor (327 amino acid residues), the algorithm cannot be used to classify mutants in Lysozyme (164 amino acid residues).

Here we identify a set of 16 predictive features that, in combination with Support Vector Machine, Random Forest, and Naïve Bayes supervised learning algorithms, avoids the overfitting problem when the training set is composed of TP53 variants and the validation set is composed of BRCA1 missense variants. Initially, we used 31 deleterious and eight neutral BRCA1 BRCT variants that had been functionally tested as our training set. However, this approach yielded poor classification performance in a cross-validation test, presumably because of small sample size (unpublished data). As an alternative, we selected our features and performed supervised learning with a training set of 600+ artificially engineered TP53 missense variants. The ability of computational learning algorithms trained on TP53 variants to classify BRCA1 missense variants in agreement with the BRCA1 functional assay (94%+) suggests that mechanisms underlying structural and functional defects may be similar in TP53 and BRCA1.

In comparison, an approach based on sequence analysis and expected frequencies of structural features inferred from mutagenesis studies of *E. Coli* lac repressor and T4 lysozyme resulted in only 75% agreement with a BRCA1 BRCT trypsin sensitivity assay of 22 variants [12,50]. We find that the best supervised learners are in greater agreement with the BRCA1 transactivation assay than several sequence analysis methods and an empirically designed set of rules and thresholds [7].

The sequence analysis methods that incorporate physiochemical properties of amino acid residues as well as evolutionary conservation (Ancestral Sequence and Align-GVGD) are more accurate than SIFT, which only considers evolutionary conservation. A weakness of these methods is that, for purposes of classifying deleterious variants, there is no principled way to choose the optimal set of evolutionarily related sequences to align and analyze. In this work, we used sets of aligned sequences taken from published work (Ancestral Sequence) [9], the SIFT and Align GVGD webservers [18,57], and a deep alignment (out to the sea urchin Strongylocentrotus purpuratus) generated by the creators of Align GVGD. Different sequence sets produce different classifications of the variants, and choice is biased by available genomes and decisions about appropriate thresholds of relatedness. The problem is illustrated with classifications of the BRCA1 BRCT missense variant V1665M. Align-GVGD Tnig, Align-GVGD Spur, and Ancestral Sequence incorrectly classify V1665M as deleterious, because valine is completely conserved in their multiple sequence alignments. In contrast, SIFT constructs an alignment that includes two *Arabidopsis* proteins containing BRCT domains (UNIPROT Q9ZWC2, Q3E7F4) with a methionine aligned at this position, and thus correctly classifies the variant as neutral.

SIFT uses *Dirichlet mixture pseudocounts* to estimate allowed amino acid residues at each alignment position [29]. Pseudocount approaches compensate for incomplete sequence sampling in a multiple sequence alignment by adding counts for imaginary amino acid residues that are statistically likely to occur at each position. Our analysis indicates that several SIFT errors are the result of poorly estimated pseudocounts (Table S1). Importantly, SIFT is the only sequence analysis method that includes an automated alignment algorithm. The accuracy of Ancestral Sequence and Align GVGD depends on manual sequence selection, so these methods cannot automatically be applied to other cancer susceptibility genes or to whole genome analysis.

Despite their limitations, sequence analysis methods have the advantage that they can be applied to any position in BRCA1, whereas our supervised learners require protein structure information and are thus limited to regions for which accurate protein structure or structural models are available. For example, the BRCA1 BRCT missense variant E1794D is misclassified by Support Vector Machine, Random Forest, Rule-based decision tree, and Decision Tree. Analysis of X-ray crystal protein structure quality with MolProbity and PROCHECK [58,59] indicates that in the 1t29 structure, the atomic coordinates at this position, may be incorrect. The corresponding backbone dihedral angles Φ and Ψ of 143.8° and 112.9°, respectively, are statistical outliers. Using sequence analysis alone, with multiple sequence alignments in which *Tetraodon* BRCA1 contains an aspartic acid residue at this position, Align-GVGD, Ancestral Sequence, and SIFT correctly classify the variant as neutral.

The empirically designed rules (Rule-based decision tree) are more accurate than a Decision Tree of rules learned by a supervised algorithm but not as accurate as Support Vector Machine, Random Forest, Naïve Bayes, Ancestral Sequence, or Align-GVGD (Table 1 and Figure 1). It appears that it is difficult to correctly set thresholds and weigh the relative importance of empirically designed rules. For example, Rule-based decision tree classifies A1669S as deleterious, in disagreement with the functional assay (Figure 1 and Table S1). Rule-based decision tree uses a “mutation likelihood” rule that would classify A1669S as neutral because there is a serine in frog *BRCA1* at this position. It also uses a rule that would classify A1669S as deleterious because the amino acid residue position is buried in the protein core and close to a buried charged residue. The order is such that the latter rule dominates the final decision. In contrast, the supervised learning approach makes no a priori decisions about thresholds or predictor ordering, but learns this information implicitly during the training process. Given an informative training set, such as the TP53 variants used in the present work, it is able to make highly accurate decisions about the BRCA1 variants.

The supervised learning algorithms described here make classification decisions based on nonlinear combinations of predictive features and fail to provide rationalizations that can be understood by humans. To address this issue, we apply a post-predictive step in which we analyze protein structure models and alignments. For example, the wild-type arginine amino-acid residue at position 1753 of BRCA1 forms a salt bridge with the glutamate at position 1735. The arginine is found in the linker region that connects the BRCT-N and BRCT-C domains and the glutamate is found in BRCT-N (Figure 4). This charge–charge interaction may be important for the stability of the BRCT homodimer. In our structural model of the threonine variant, the salt bridge is broken, potentially destabilizing the homodimer. Both R1753 and E1735 are completely conserved in our alignment of 13 BRCA1 orthologs (Figure 5), suggesting possible selective pressure to preserve their pairwise interaction. Such rationalizations increase our confidence in the predictions and suggest ways to test them experimentally—for example, by site-directed mutagenesis. Fifty-four percent of the variants can be explained by combining structural and evolutionary analysis in post-prediction (Tables S1 and S2).

### Binding Site

Several studies of the BRCA1 BRCT domains have suggested that there may be surface patches that interact with protein partners [60–63]. In previous work, we predicted that a groove formed by both BRCT repeats (near nonfunctional variants L1657 and K1702) and the ridge that delimits the groove (near nonfunctional variant E1660) constitutes such a surface patch [7]. Our prediction was subsequently confirmed through X-ray crystallographic studies, which revealed a site where the phosphorylated peptides of BACH1 and CtIP have been found to bind [64,65]. Importantly, several missense variants were found to disrupt this interaction [64,65], suggesting that clustering of deleterious variants at solvent-exposed amino acid residue positions is indeed a useful indicator of binding site location. There is a large literature on the general topic of the relationship between deleterious mutations and binding sites [66–74].

Two surface variants found to be deleterious to BRCA1 transactivation activity in our companion paper (R1753T and T1685I) [14] lie on a highly conserved patch of amino acid residues, forming an exposed groove. The R1753T variant yields a changed electrostatic surface potential, which may be sufficient to disrupt the binding of BRCA1 to a protein partner or nucleic acid ligand important for transactivation. Following the logic that predicted the BACH1/ CtIP binding site, we suggest that this groove may be a previously uncharacterized binding site, whose disruption inactivates BRCA1 transactivation function. Accordingly, we are currently testing the binding of several candidate protein partners to the predicted binding site using site-directed mutagenesis and a yeast two-hybrid assay.

### Prioritizing Variants for Biochemical Testing

Biochemical assays can play an important role in identifying deleterious UCVs in cancer susceptibility genes [75], but the work is labor-intensive and time-consuming. We estimate that, on average, an assay for one BRCA1 UCV in a mammalian cell system costs US$125–US$150 and requires three weeks of personnel time, from ordering primers to final results. The time can be reduced by processing the variants in batches. To assay the 54 uncharacterized BRCT BRCA1 missense variants found in the BIC database (April 2006) would take approximately 18 months of personnel time. Accurate computational classification of UCVs can significantly reduce the required time by prioritizing UCVs most likely to be deleterious. Importantly, while computational classification and functional assays can contribute to medical decision making, other factors such as family history, co-occurrence with known deleterious mutations, and studies of patient tumor tissue will continue to be important in a clinical setting.

### Generalizability of the Methods

We have applied supervised learners trained on the TP53 variant set to prediction of UCVs in BRCA2, with promising results (unpublished data). We are currently exploring whether this training set and our current set of features can be used to evaluate UCVs in other genes associated with familial cancer syndromes: MLH1, MSH2, MSH6 (hereditary nonpolyposis colon cancer), APC (familial adenomatous polyposis), MYH (MYH adenomatous polyposis), and P16 (melanoma). We have applied a modified version of this method to classify all human amino-acid changing SNPs found in the dbSNP database [76] as deleterious or neutral [56]. The SNPs were classified with a support vector machine trained on amino acid residue substitutions from more than 1,500 human proteins. Because X-ray crystal structures are not available for most human proteins [77], we built homology models with an automated modeling pipeline MODPIPE that relies on the MODELLER package for fold assignment, sequence-structure alignment, model building, and model assessment [78]. A small number of these predictions have been validated by biochemical and epidemiological studies found in the literature.

We are exploring the extent to which a decision rule learned with a training set of variants from one protein, such as TP53, can be generalized to variants from other proteins. One possibility is that most deleterious missense mutants do not affect specific binding interactions, but are instead slightly destabilizing [79]. If this is true, a training set of missense variants from a protein with similar stability to the protein of interest may be the best choice. Other possiblities include training on a protein sharing GO terms, or from the same fold family (all-alpha, all-beta, alpha-beta, etc.) as the protein of interest. We are working on generating large variant datasets from selected proteins to test these hypotheses.

In summary, we have systematically and comprehensively evaluated structure- and sequence-based computational prediction methods applied to variants in the BRCA1 BRCT domains and developed detailed structural explanations for the measured and predicted impact of 49 BRCA1 variants. When combined with 16 carefully selected predictive features, the best-supervised learning algorithms are in greater agreement with experimental results than has been reported previously.

The increased use of sequencing methods to genotype individuals at risk for inherited cancers and the observation that sequence variation is greater in ethnic minorities than in Caucasians highlight the need for improved methods of UCV risk assessment. Bioinformatics approaches including supervised learning algorithms, protein structure modeling, and evolutionary sequence analysis can contribute to an integrated approach to risk assessment by increasing coverage of classified UCVs more rapidly than is possible by functional assays. In the future, when clinicians counsel patients about their cancer risk, they will be able to take advantage of these bioinformatics prediction methods. Finally, successful generalization of these methods to a large number of disease-associated genes will play an important role in reducing the growing number of loci, variants, and phenotypes that confound modern whole genome disease-association studies.

**Table 3:**
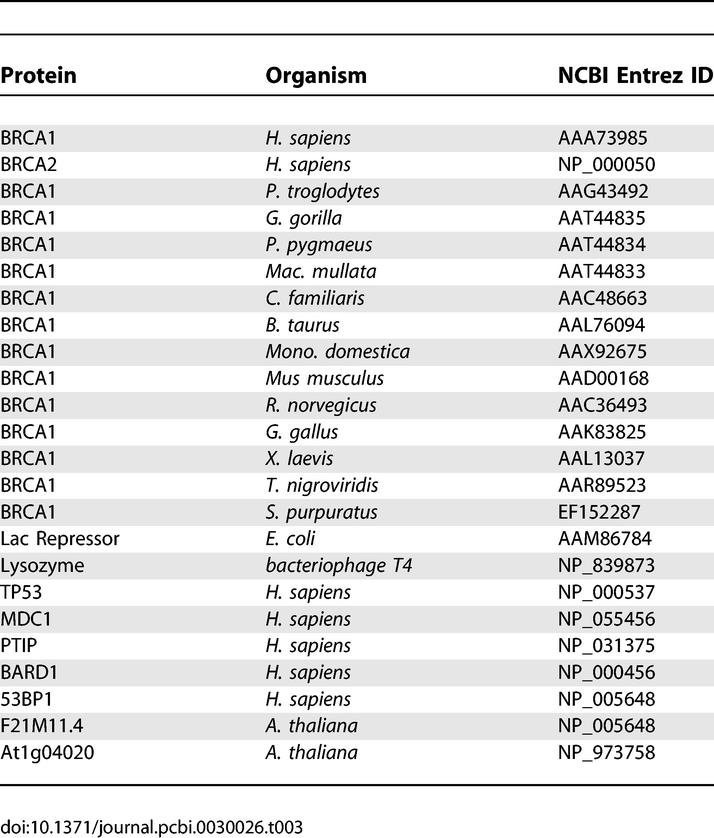
Accession Numbers

## Supporting Information

Table S1 Post-Prediction Analysis of BRCA1 BRCT Missense Variants Characterized by Transactivation Assays Transcription assay in yeast (Y) or mammalian (M) cells. We used a 50% activity cutoff value. ○, neutral/low clinical relevance; •, deleterious/high-risk variant; red, variant names are colored red if classification considering both yeast and mammalian assays is deleterious/high-risk, blue, variant names are colored blue if classification considering both yeast and mammalian assays are neutral/low clinical relevance. There is no available post-prediction analysis for S1715R, T1720A, and P1806A.
(135 KB DOC)

Table S2 Structural Explanations for the Impact of BRCA1 BRCT UCVs Found in BIC (Excluding Those in Table S1 and Those for Which Definitive Genetic Evidence Exists) Consensus Classification is majority vote of Random Forest, Naïve Bayes, and Support Vector Machine classifiers. Red, deleterious; blue, neutral.
(101 KB DOC)

Table S3 Core Set of 13 Predictive Features Identified Using a Mutual Information Analysis The mutual information between each feature and the functional/nonfunctional class of each TP53 missense mutant was computed as described previously [24].
(50 KB DOC)

Table S4 Predictive Features of Missense Mutants That Were Tried but Failed To Improve Support Vector Machine Performance on the TP53 Training Set These were not included in the optimal 16 features selected for use with the supervised learning algorithms.
(40 KB DOC)

Table S5 Performance of Optimal 16 Features Evaluated with 10-Fold Cross-Validation Using the TP53 Missense Variant Training Set Performance was computed with all 16 features, then with each of the 16 features held out. MCC, Matthews correlation coefficient; TNR, true negative rate; TPR, true positive rate.
(63 KB DOC)

### Accession Numbers

Accession numbers from the National Center for Biotechnology Information (http://www.ncbi.nlm.nih.gov) are shown in Table 3.

## Abbreviations

Align-GVGD: Align Grantham Variation Grantham Deviation
AUC: area under the ROC curve
BIC: breast information core database
BRCT: BRCA1 C-terminal domains
BRCT-C: BRCT C-terminal domain
BRCT-N: BRCT N-terminal domain
GD: Grantham Deviation
GV: Grantham Variation
ROC: receiver operating characteristic
Rule-based decision tree: empirically derived rules encoded in a decision tree
SIFT: Sorting Intolerant from Tolerant
UCV: variant of unknown clinical significance
