# Supplementary material for: Functional Impact of Missense Variants in BRCA1 Predicted by Supervised Learning
Source: PLoS Comput Biol. 2007 Feb 16;3(2):e26. doi: 10.1371/journal.pcbi.0030026 (PMC1797820; doi:10.1371/journal.pcbi.0030026)
Supplement: Supplementary file 1 [file pcbi.0030026.st001.doc]

**Table S1**. **Post-prediction analysis of BRCA1 BRCT missense variants characterized by transactivation assays.** ^a^Transcription assay in yeast (Y) or mammalian (M) cells. We used a 50% activity cut-off value. “⭘” neutral/low clinical relevance; “⚫“ deleterious/high-risk variant; Variant names are colored red if classification considering both yeast and mammalian assays is deleterious/high-risk and blue if neutral/low clinical relevance. There is no available post-prediction analysis for S1715R, T1720A and P1806A.

| **Exon** | **Variant** | **TNX Y^a^** | **TNX M** | **Comments** |
| --- | --- | --- | --- | --- |
| 16 | **S1655F** | ⚫ | ⚫ | Defective in phosphopeptide binding assay [1]. F breaks the hydrogen bond between S1655 (in β1) and T1700 (in α2) in BRCT-N, which may destabilize the protein. F creates steric clashes with the phosphoserine SEP6 in BACH1, a key residue in BRCA1-BACH1 binding. |
| 17 | **L1664P** | ⭘ | ⭘ | Correctly classified only by Naïve Bayes, Align GVGD, and Ancestral Sequence. Align GVGD classifies it as "Enriched Neutral 2" (Methods). Ancestral Sequence classifies it correctly because in mammalian BRCA1 orthologs, a variety of amino acid residues appear in this position (although never a P). P occurs in second turn of the α1 helix, where in general, it would be expected to destabilize the protein. However, the α1 helix is distant from the phosphopeptide binding site, our proposed new binding site, and the tandem BRCT domain interface. Possibly the destabilization of this part of the protein does not impair transactivation. |
| 17 | **V1665M** | ⭘ | ⭘ | V is at a buried position in the in α1 helix and completely conserved in multiple sequence alignments. SIFT includes two *Arabidopsis* proteins with BRCT domains (Q9ZWC2 and Q3E7F4) having M aligned at this position. The rationalization for the incorrect Rule-based Decision Tree classification of deleterious is "buried and buried charge at a neighboring position". The local environment is hydrophobic, tolerating both V and M. Introduction of M does not induce substantial strain on the model. |
| 17 | **A1669S** | ⭘ | ⭘ | A is at a buried position in the in α1 helix. Incorrectly classified as deleterious by Ancestral Sequence which uses an alignment of mammalian BRCA1 orthologs in which A is completely conserved. Alignments used by all other methods include an ortholog from frog with S aligned at this position. The rationalization for the incorrect Rule-based Decision Tree classification of deleterious is "buried and buried charge at a neighboring position". Although the substitution introduces a small polar residue into a hydrophobic environment in the protein core, the partial negative charge in the serine γ oxygen atom may be satisfied by forming a hydrogen bond with the carbonyl oxygen of V1665. |
| 17 | **T1685I** | ⚫ | ⚫ | T is at an exposed backbone turn and is part of a hydrogen bonding network found in BRCT-N where we predict a novel binding site. |
| 17 | **M1689R** | ⚫ | ⚫ | Introduces a buried charge, likely destabilizing the hydrophobic core. |
| 18 | **C1697R** | ⚫ | ⚫ | Introduces a buried charge, likely destabilizing the protein's hydrophobic core. |
| 18 | **R1699W** | ⭘ | ⚫ | Located at the predicted binding site for RNA pol II and participates in a salt bridge that stabilizes the interaction between both BRCT domains [2,3]. Activity is reduced (<40% of wt) in mammalian cells. Note that activity is comparable to wt BRCA1 in yeast cells. This variant has been shown to be a temperature-sensitive mutant [3]. In cases where temperature-sensitivity is at issue we classify the variant according to the results obtained at the temperature corresponding to the in vivo condition (37°C). |
| 18 | **R1699Q** | ⭘ | ⚫ | Located at the predicted binding site for RNA pol II and participates in a salt bridge that stabilizes the interaction between both BRCT domains [2,3]. Activity is reduced (<40% of wt) in mammalian cells. Note that activity is comparable to wt BRCA1 in yeast cells. This variant also shows characteristics of a temperature-sensitive mutant. |
| 18 | **T1700A** | ⚫ | ⚫ | T may play a key role in BRCA1 binding to phosphorylated protein partners. In the BRCA1-BACH1 peptide holo structure (1t29), T1700 is close to BACH1 and involved in a hydrogen bond network with the phosphorylated serine (SEP6) in BACH1. Threonine's sidechain oxygen OG1 hydrogen bonds to a serine OG1 oxygen (S1655). The serine OG1 hydrogen bonds to SEP6. |
| 18 | **G1706E** | ⚫ | ⚫ | In the α2 helix of BRCT-N which contains residues critical for binding with BACH1 in the 1t29 structure. Situated between two lysines (K1702 and K1711). K1702 forms a hydrogen bond with the phosphoserine, the Z nitrogen of K1711 likely forms hydrogen bonds with the backbone carbonyl oxygens of T1681 and T1684. The glutamate introduces negative charge in a completely buried position and could destabilize the hydrogen bonds of the neighboring lysines. |
| 18 | **G1706A** | ⭘ | ⚫ | SIFT incorrectly classifies it as neutral because the SIFT alignment contains two rice (*Oryza sativa)* proteins that contain putative BRCT domains (Q5TKH9, Q6L532) with A aligned at this position. The proteins are not in alignments used by other methods, which have G completely conserved. Showed a slightly reduced activity in yeast (~65%) but a markedly decrease of activity in mammalian cells (<10%) [4]. One possibility that has not been investigated is that this variant is also a temperature-sensitive variant. Lovelock et al. performed a transcription activation assay and found the variant to have activity comparable to wild type [5]. |
| 18 | **A1708E** | ⚫ | ⚫ | SIFT incorrectly classifies it as neutral. The SIFT alignment uses Dirichlet mixture pseudocounts to estimate frequencies of amino acid residues not observed in an alignment column and rationalizes that E is likely to appear aligned at this position, although E is not observed at this position in the SIFT alignment. |
| 18 | **V1713A** | ⚫ | ⚫ | Lies in the β4 strand of the 4-stranded parallel beta sheet in BRCT-N. In BRCT tandem domains, hydrophobic, β-branched amino acid residues are conserved at this position. β-branched amino acid residues are generally favored in β-sheets. A is not β-branched |
| 18 | **S1715N** | ⚫ | ⚫ | SIFT incorrectly classifies it as neutral because the SIFT alignment includes two *Arabidopsis* proteins that contain BRCT domains (Q9ZWC2 Q3E7F4) having N aligned at this position. S is completely conserved in multiple sequence alignments used by the other methods. |
| 19 | **W1718C** | ⚫ | ⚫ | W is a large, hydrophobic amino acid residue, buried in the core of BRCT-N. Replacement by C is likely destabilizing. |
| 20 | **V1736A** | ⚫ | ⚫ | Activity comparable to the M1775R deleterious control in the small colony phenotype assay [6]. |
| 20 | **G1738R** | ⚫ | ⚫ | Introduces a large positively charged residue in a buried position. Likely destabilizing. Defective in phosphopeptide binding assay. Severe folding defect found in trypsin sensitivity assay [1]. |
| 20 | **G1738E** | ⚫ | ⚫ | Introduces negatively charged residue in a buried position. Likely destabilizing. |
| 20 | **A1752P** | ⚫ | ⚫ | In linker region connecting the two BRCT domains.Incorrectly classified as neutral by Align-GVGD which takes into account amino acid sidechain composition, polarity, and volume. As there is no consideration of bond angles, it is vulnerable to miscalling substitutions to P. |
| 20 | **R1753T** | ⚫ | ⚫ |  Located in a linker region connecting the two BRCT domains at a predicted binding site. R1753 forms a salt bridge with E1735 which is in the N-terminal BRCT domain. The salt bridge is broken when R is replaced by T. |
| 21 | **L1764P** | ⚫ | ⚫ |  L is a key conserved hydrophobic core residue characteristic of the BRCT superfamily [1]. |
| 21 | **I1766S** | ⚫ | ⚫ | Completely buried position on beta strand β1' in hydrophobic core. Introduction of S creates buried unsatisfied hydrogen bond donor/acceptor at the S γ oxygen. |
| 21 | **M1775R** | ⚫ | ⚫ | M1775 is part of the hydrophobic binding pocket where the key phosphopeptide binding residue F binds to BRCA1. Substitution by a positively charged residue such as R would reduce/abolish binding affinity of BRCA1 and phosphopeptide. Incorrectly classified as neutral by SIFT. The SIFT alignment uses Dirichlet mixture pseudocounts to estimate frequencies of amino acid residues not observed in an alignment column and rationalizes that R is likely to appear aligned at this position, although R is not observed at this position in the SIFT alignment. |
| 21 | **M1775K** | ⚫ | ⚫ | Incorrectly classified as neutral by SIFT. The SIFT alignment uses Dirichlet mixture pseudocounts to estimate frequencies of amino acid residues not observed in an alignment column and rationalizes that K is likely to appear aligned at this position, although K is not observed at this position in the SIFT alignment. Substitution by a positively charged residue such as K would reduce/abolish binding affinity of BRCA1 and phosphopeptide. Markedly reduced activity in yeast and mammalian cells (Foulkes et al. unpublished). |
| 21 | **Q1785H** | ⭘ | ⭘ | Q is on the exposed, hydrophilic side of amphipathic helix α1' and sticks out into solvent. It makes no sidechain hydrogen bonds with any other residue. H is also hydrophilic and should be compatible with this position. There is a H in the equivalent position of the dog BRCA1 sequence in our sequence alignment. |
| 22 | **G1788D** | ⚫ | ⚫ | The protein backbone makes a tight turn at this position which requires a G. D disrupts the BRCT superfamily conserved G[G/A] motif [7]. |
| 22 | **G1788V** | ⚫ | ⚫ | V disrupts the BRCT superfamily conserved G[G/A] motif [7]. |
| 22 | **E1794D** | ⭘ | ⭘ | Located at an exposed site in a backbone bend position. Replacement of one negatively charged amino acid residue for another. Incorrectly rationalized as deleterious by four of the five methods that consider protein structure. Atomic coordinates of this position are likely incorrect in 1t29 crystal structure. According to several crystal structure quality analysis programs, the backbone angles at this position are statistical outliers (Discussion). Our *in silico* mutation procedure had great difficulty placing D1794, the final model contains considerable strain. A D is found in *Tetraodon* BRCA1 at the equivalent position in our alignment of orthologs. |
| 23 | **V1804D** | ⭘ | ⭘ | Located in an exposed loop. The introduction of a charged residue (D) on the protein surface is not likely to impact function unless it is at a binding site where the charge is unfavorable. Here, position 1804 is in a surface area that is not conserved in our alignment. SIFT correctly classifies it as neutral. The SIFT alignment uses Dirichlet mixture pseudocounts to estimate frequencies of amino acid residues not observed in an alignment column and rationalizes that D is likely to appear aligned at this position, although D is not observed at this position in the SIFT alignment. Activity comparable to wild type in the small colony phenotype in yeast [6]. |
| 23 | **V1809F** | ⚫ | ⚫ | The rationalization for the incorrect Rule based Decision Tree classification of neutral is "likely amino acid substitution". |
| 24 | **W1837R** | ⚫ | ⚫ | In BRCT fold conserved WXXX[S/C] motif [7]. |

**References**

1. Williams RS, Lee MS, Hau DD, Glover JN (2004) Structural basis of phosphopeptide recognition by the BRCT domain of BRCA1. Nat StructMolBiol 11: 519-525.

2. Vallon-Christersson J, Cayanan C, Haraldsson K, Loman N, Bergthorsson JT, et al. (2001) Functional analysis of BRCA1 C-terminal missense mutations identified in breast and ovarian cancer families. Hum MolGenet 10: 353-360.

3. Worley T, Vallon-Christersson J, Billack B, Borg A, Monteiro AN (2002) A Naturally Occurring Allele of BRCA1 Coding for a Temperature-Sensitive Mutant Protein. Cancer BiolTher 1: 497-501.

4. Phelan CM, Dapic V, Tice B, Favis R, Kwan E, et al. (2005) Classification of BRCA1 missense variants of unknown clinical significance. J Med Genet 42: 138-146.

5. Lovelock PK, Healey S, Au W, Sum E, Tesoriero A, et al. (2005) Genetic, functional and histopathological evaluation of two C-terminal BRCA1 missense variants. J Med Genet: 74-83.

6. Coyne RS, McDonald HB, Edgemon K, Brody LC (2004) Functional Characterization of BRCA1 Sequence Variants Using a Yeast Small Colony Phenotype Assay. Cancer BiolTher 3.

7. Zhang X, Morera S, Bates PA, Whitehead PC, Coffer AI, et al. (1998) Structure of an XRCC1 BRCT domain: a new protein-protein interaction module. EMBO J 17: 6404-6411.
