# Supplementary material for: Functional Impact of Missense Variants in BRCA1 Predicted by Supervised Learning
Source: PLoS Comput Biol. 2007 Feb 16;3(2):e26. doi: 10.1371/journal.pcbi.0030026 (PMC1797820; doi:10.1371/journal.pcbi.0030026)
Supplement: Supplementary file 2 [file pcbi.0030026.st002.doc]

**Table S2. Structural explanations for the impact of BRCA1 BRCT UCVs found in BIC** (excluding those in Supplementary Table 1 and those for which definitive genetic evidence exists). Consensus Classification is majority vote of Random Forest, Naïve Bayes, and Support Vector Machine classifiers. Red=Deleterious. Blue=Neutral.

| **Exon** | **Variant** | **Consensus Classification** | **Comments on structure and results from alternative methods** |
| --- | --- | --- | --- |
| 16 | M1652T | D | A key conserved hydrophobic core residue characteristic of the BRCT superfamily [1]. The polar threonine disrupts hydrophobicity at this position. |
| 16 | V1653M | D | Lies in the β1 strand of the 4-stranded parallel beta sheet in BRCT-N. In BRCT tandem domains, hydrophobic, beta-branched amino acid residues are conserved at this position. Beta-branched amino acid residues are generally favored in beta sheets. M is not beta-branched and may cause steric clashes with V1687, its nearest neighbor which lies in the β3 strand. |
| 17 | T1685A | D | Located at a putative binding site. |
| 17 | T1691I | D | T1691 could form a hydrogen bond with S1715 (in β4). Bond would be broken by its replacement by I, leaving an unsatisfied hydrogen donor/acceptor in a buried position. MODELLER has difficulty eliminating strain from the model when I is substituted here. |
| 18 | D1692Y | D | Exposed salt-bridge with R1744 is broken by Y replacement, possible impact on stability (although not as destabilizing as a broken buried salt-bridge). MODELLER has difficulty eliminating strain from the model when Y is substituted here. Defective in phosphorylated peptide binding (pSer-X-X-Phe) assay. Intermediate folding defect found in trypsin sensitivity assay [1] . |
| 18 | F1695L | D | Specifically binds phosphorylated peptide (pSer-X-X-Phe and not Ser-X-X-Phe). Stability equivalent to wild-type in trypsin sensitivity assay [1]. |
| 18 | V1696L | D | V1696 forms a small pocket in the protein surface and I1 of the BACH1 peptide in 1t29 lies in the pocket. The L replacement makes subtle changes in the shape of this pocket and may weaken the hydrophobic interaction between BRCA1 and BACH1. Defective in phosphorylated peptide binding (pSer-X-X-Phe) assay. Intermediate folding defect found in trypsin sensitivity assay [1]. |
| 18 | S1715C | D | S1715 (β4) forms hydrogen bonds with many neighboring amino acid residues, including T1691, F1717 (α3), W1718 (α3 and in BRCT WXXX[S/C] signature motif [2]), and F1734. The variant C can only form a hydrogen bond with W1718. Likely destabilizing. |
| 19 | S1722F | D | Lies in a neighborhood with many large, completely buried aromatic amino acid residues (F1717, W1718, F1668). Introduction of another large aromatic (F1722) in a completely buried position likely causes steric conflicts and hydrophobic core destabilization. |
| 20 | F1734S | D | Replacement of large aromatic residue by a small polar residue in a neighborhood where there are no available hydrogen bonding partners. The S will have unfulfilled hydrogen bond donors/acceptors in a completely buried position. Likely destabilizing. |
| 20 | V1736G | D | V1736 is a key conserved hydrophobic core residue characteristic of the BRCT superfamily [1] . |
| 20 | R1751Q | D | In αL helix in the linker region connecting the two BRCT domains, known to be sensitive to mutation [1]. Specifically binds phosphorylated peptide (pSer-X-X-Phe and not Ser-X-X-Phe). Intermediate folding defect found in trypsin sensitivity assay [1]. |
| 20 | R1751P | D | In αL helix in the linker region connecting the two BRCT domains known to be sensitive to mutation [1]. The proline occurs in the second turn of the helix where it forms a kink and is destabilizing. |
| 22 | L1780P | D | Loss of transactivation function in yeast [3]. |
| 22 | M1783T | D | Specifically binds phosphorylated peptide (pSer-X-X-Phe and not Ser-X-X-Phe). Intermediate folding defect found in trypsin sensitivity assay [1]. |
| 22 | A1789S | D | S disrupts the BRCT superfamily conserved G[G/A] motif [2]. |
| 23 | V1808A | D | In BRCT tandem domains, hydrophobic, beta-branched amino acid residues are conserved at this position (also in BRCA1 orthologs). Beta-branched amino acid residues are energetically favored in beta sheets. A is not beta-branched. Likely destabilizing. |
| 23 | V1809A | D | In BRCA1 orthologs, hydrophobic, beta-branched amino acid residues are conserved at this position. Beta-branched amino acid residues are energetically favored in beta sheets. A is not beta-branched. Likely destabilizing. |
| 23 | V1810G | D | In BRCA1 orthologs, hydrophobic, beta-branched amino acid residues are conserved at this position. Beta-branched amino acid residues are energetically favored in beta sheets. G is neither beta-branched nor hydrophobic. Likely destabilizing |
| 24 | W1837G | D | Disrupts the highly conserved BRCT superfamily WXXX[S/C] motif [2] Defective in phosphorylated peptide binding (pSer-X-X-Phe) assay. Severe folding defect found in trypsin sensitivity assay [1]. |
| 24 | V1838E | D | V1838 is a key conserved hydrophobic core residue characteristic of the BRCT superfamily [1]. |
| 24 | S1841N | D | Disrupts the highly conserved BRCT superfamily WXXX[S/C] motif [2]. |
| 24 | A1843P | D | The proline occurs in the third turn of the helix where it forms a kink and is destabilizing. |
| 24 | L1844R | D | Introduces large positively charged residue in a buried position. |
| 24 | Y1853C | D | Hydroxyl group of Y1853 forms hydrogen bond with carboxyl oxygen of D1840 (found in the WXXX[C/S] motif), and the C variant breaks the bond. Defective in phosphorylated peptide binding (pSer-X-X-Phe) assay. Severe folding defect found in trypsin sensitivity assay [1]. |

**References**

1. Williams RS, Lee MS, Hau DD, Glover JN (2004) Structural basis of phosphopeptide recognition by the BRCT domain of BRCA1. Nat StructMolBiol 11: 519-525.

2. Zhang X, Morera S, Bates PA, Whitehead PC, Coffer AI, et al. (1998) Structure of an XRCC1 BRCT domain: a new protein-protein interaction module. EMBO J 17: 6404-6411.

3. Hayes F, Cayanan C, Barilla D, Monteiro AN (2000) Functional assay for BRCA1: mutagenesis of the COOH-terminal region reveals critical residues for transcription activation. Cancer Res 60: 2411-2418.
