# Supplementary material for: Functional Impact of Missense Variants in BRCA1 Predicted by Supervised Learning
Source: PLoS Comput Biol. 2007 Feb 16;3(2):e26. doi: 10.1371/journal.pcbi.0030026 (PMC1797820; doi:10.1371/journal.pcbi.0030026)
Supplement: Supplementary file 3 [file pcbi.0030026.st003.doc]

**Table S3. Core set of 13 predictive features identified using a mutual information analysis.** The mutual information between each feature and the Functional/Non-functional class of each TP53 missense mutant was computed as described previously [1].

| **Feature** | **Mutual Information (Bits)** |
| --- | --- |
| Solvent Accessiblity of wild-type amino acid residue (Å^2^) | 0.146 |
| Relative entropy estimated by amino acids in the variant's alignment column [2] | 0.145 |
| Solvent Accessibility of variant residue | 0.142 |
| Solvent Accessibility of wild-type residue normalized by maximum exposed Solvent Accessibility of that residue type in a GLY-X-GLY tripeptide, using values given by Rose *et al*. [3] | 0.132 |
| Normalized Solvent Accessibility of variant residue | 0.122 |
| Positional hidden Markov model conservation score based on the probabilities of the wild-type, variant, and most probable amino acid residue in the variant’s alignment column [1] | 0.081 |
| Number of unsatisfied spatial restraints in the MODELLER objective function after *in silico* mutation and simulated annealing refinement of the variant. Two thresholds are used to identify violated restraints yielding two features. | 0.043 |
| Change in volume (Å^3^) [4] | 0.042 |
| Grantham difference [5] | 0.035 |
| Whether the mutation results in buried charge | 0.031 |
| Change in polarity [6] | 0.016 |
| Change in formal charge | 0.0078 |

**References**

1. Karchin R, Kelly L, Sali A (2005) Improving functional annotation of non-synonomous SNPs with information theory. Pac Symp Biocomput: 397-408.

2. Kullback S (1959) Information theory and statistics. New York: Wiley.

3. Rose GD, Geselowitz AR, Lesser GJ, Lee RH, Zehfus MH (1985) Hydrophobicity of amino acid residues in globular proteins. Science 229: 834-838.

4. Zamyatnin AA (1972) Protein volume in solution. Prog Biophys Mol Biol 24: 107-123.

5. Grantham R (1974) Amino acid difference formula to help explain protein evolution. Science 185: 862-864.

6. Engelman DM, Steitz TA, Goldman A (1986) Identifying nonpolar transbilayer helices in amino acid sequences of membrane proteins. Annu Rev Biophys Biophys Chem 15: 321-353.
