# Supplementary material for: Functional Impact of Missense Variants in BRCA1 Predicted by Supervised Learning
Source: PLoS Comput Biol. 2007 Feb 16;3(2):e26. doi: 10.1371/journal.pcbi.0030026 (PMC1797820; doi:10.1371/journal.pcbi.0030026)
Supplement: Supplementary file 4 [file pcbi.0030026.st004.doc]

**Table S4**.  **Predictive features of missense mutants that were tried but failed to improve support vector machine performance on the TP53 training set.** These were not included in the optimal 16 features selected for use with the supervised learning algorithms**.**

| **Feature** | **Description** |
| --- | --- |
| Aliphatic wild-type | Wild-type amino acid has only carbon and hydrogen atoms (I,V,L,A,P) or is a methionine |
| Aliphatic variant | Variant amino acid has only carbon and hydrogen atoms (I,V,L,A,P) or is a methionine |
| Tiny wild-type | Wild-type amino acid is very small (G,A,S,P) |
| Tiny variant | Variant amino acid is very small (G,A,S,P) |
| Positively charged wild-type | Wild-type amino acid has a positive formal charge at physiological temperature (R,K) |
| Positively charged variant | Variant amino acid has a positive formal charge at physiological temperature (R,K) |
| Negatively charged wild-type | Wild-type amino acid has a negative formal charge at physiological temperature (D,E) |
| Negatively charged variant | Variant amino acid has a negative formal charge at physiological temperature (D,E) |
| Proline wild-type | Wild-type amino acid is proline |
| Proline variant | Variant amino acid is proline |
| Hydrophobic wild-type | Wild-type amino acid is hydrophobic (I,V,L,M,F,W,C) |
| Hydrophobic variant | Variant amino acid is hydrophobic (I,V,L,M,F,W,C) |
| C-beta branched wild-type | Wild-type amino acid is C-beta branched (T,V,I) |
| C-beta branched variant | Variant amino acid is C-beta branched (T,V,I) |
| Change in hydrogen bond number | Change in number of hydrogen bonds after variant is substituted into protein structure and its position is refined with molecular dynamics |
