# Supplementary material for: Functional Impact of Missense Variants in BRCA1 Predicted by Supervised Learning
Source: PLoS Comput Biol. 2007 Feb 16;3(2):e26. doi: 10.1371/journal.pcbi.0030026 (PMC1797820; doi:10.1371/journal.pcbi.0030026)
Supplement: Supplementary file 5 [file pcbi.0030026.st005.doc]

**Table S5**.  **Performance of optimal 16 features evaluated with 10-fold cross-validation using the TP53 missense variant training set.**  Performance was computed with all 16 features, then with each of the 16 features held out. MCC=Matthews correlation coefficient. TPR=true positive rate. TNR=true negative rate.

| **Feature set** | **Held out feature** | **MCC** | **TPR** | **TNR** |
| --- | --- | --- | --- | --- |
| 16 | None | 0.701 | 0.918 | 0.768 |
| 15 | Solvent Accessiblity of wild-type amino acid residue (Å^2^) | 0.670 | 0.893 | 0.772 |
| 15 | Solvent Accessibility of wild-type residue normalized by maximum exposed Solvent Accessibility of that residue type in a GLY-X-GLY tripeptide, using values given by Rose *et al*. [1] | 0.621 | 0.876 | 0.740 |
| 15 | So<lvent Accessibility of variant residue | 0.664 | 0.881 | 0.783 |
| 15 | Normalized Solvent Accessibility of variant residue | 0.614 | 0.831 | 0.795 |
| 15 | Number of methyl(ene) groups within 6 Å of the variant sidechain [2] | 0.700 | 0.915 | 0.772 |
| 15 | Number of unsatisfied spatial restraints in the MODELLER objective function after *in silico* mutation and simulated annealing   refinement of the variant | 0.662 | 0.885 | 0.774 |
| 15 | Standardized count of unsatisfied spatial restraints in the MODELLER objective function after *in silico* mutation and simulated annealing   refinement of the variant | 0.637 | 0.891 | 0.735 |
| 14 | Φ and Ψ backbone dihedral angle at the mutated position | 0.632 | 0.884 | 0.741 |
| 15 | Whether the mutation results in buried charge | 0.680 | 0.896 | 0.779 |
| 15 | Change in formal charge | 0.639 | 0.877 | 0.759 |
| 15 | Change in volume (Å^3^) [3] | 0.663 | 0.891 | 0.766 |
| 15 | Change in polarity [4] | 0.665 | 0.889 | 0.772 |
| 15 | Grantham difference [5] | 0.660 | 0.907 | 0.737 |
| 15 | Relative entropy estimated by amino acids in the variant's alignment column [6] | 0.654 | 0.904 | 0.735 |
| 15 | Positional hidden Markov model conservation score based on the probabilities of the wild-type, variant, and most probable amino acid residue in the variant’s alignment column [7] | 0.636 | 0.889 | 0.738 |

**References**

1. Rose GD, Geselowitz AR, Lesser GJ, Lee RH, Zehfus MH (1985) Hydrophobicity of amino acid residues in globular proteins. Science 229: 834-838.

2. Otzen DE, Rheinnecker M, Fersht AR (1995) Structural factors contributing to the hydrophobic effect: the partly exposed hydrophobic minicore in chymotrypsin inhibitor 2. Biochemistry 34: 13051-13058.

3. Zamyatnin AA (1972) Protein volume in solution. Prog Biophys Mol Biol 24: 107-123.

4. Engelman DM, Steitz TA, Goldman A (1986) Identifying nonpolar transbilayer helices in amino acid sequences of membrane proteins. Annu Rev Biophys Biophys Chem 15: 321-353.

5. Grantham R (1974) Amino acid difference formula to help explain protein evolution. Science 185: 862-864.

6. Kullback S (1959) Information theory and statistics. New York: Wiley.

7. Karchin R, Kelly L, Sali A (2005) Improving functional annotation of non-synonomous SNPs with information theory. Pac Symp Biocomput: 397-408.
